# “Struggling with practices” – a qualitative study of factors influencing the implementation of clinical quality registries for cardiac rehabilitation in England and Denmark

**DOI:** 10.1186/s12913-019-3940-5

**Published:** 2019-02-06

**Authors:** Cecilie Lindström Egholm, Charlotte Helmark, Patrick Doherty, Per Nilsen, Ann-Dorthe Zwisler, Gitte Bunkenborg

**Affiliations:** 1 The Danish Knowledge Centre for Rehabilitation and Palliative Care, University of Southern Denmark and Odense University Hospital, Southern Region of Denmark, Vestergade 17, 5800 Nyborg, Denmark; 2 Department of Medicine, Holbaek University Hospital, Smedelundsgade 60, 4300 Holbaek, Region Zealand Denmark; 3 Department of Cardiology, Zealand University Hospital, 4000 Roskilde, Denmark; 4 The National Audit of Cardiac Rehabilitation, Department of Health Sciences, Seebohm Rowntree Building, University of York, Heslington, York, YO10 5DD UK; 5 Division of Community Medicine, Department of Medical and Health Sciences, Linköping University, Campus US, Hus 511-001, ingång 76, plan 13, 58183 Linköping, Sweden; 6 Department of Anaestesiology, Holbaek University Hospital, Smedelundsgade 60, 4300 Holbaek, Region Zealand Denmark

**Keywords:** Clinical quality registry, Clinical audit, Quality improvement, Implementation, Data entry, Cardiac rehabilitation

## Abstract

**Background:**

The use of clinical quality registries as means for data driven improvement in healthcare seem promising. However, their use has been shown to be challenged by a number of aspects, and we suggest some may be related to poor implementation. There is a paucity of literature regarding barriers and facilitators for registry implementation, in particular aspects related to data collection and entry. We aimed to illuminate this by exploring how staff perceive the implementation process related to the registries within the field of cardiac rehabilitation in England and Denmark.

**Methods:**

A qualitative, interview-based study with staff involved in collecting and/or entering data into the two case registries (England *N* = 12, Denmark N = 12). Interviews were analysed using content analysis. The Consolidated Framework for Implementation Research was used to guide interviews and the interpretation of results.

**Results:**

The analysis identified both similarities and differences within and between the studied registries, and resulted in clarification of staffs´ experiences in an overarching theme: ´Struggling with practices´ and five categories; the data entry process, registry quality, resources and management support, quality improvement and the wider healthcare context. Overall, implementation received little focused attention. There was a lack of active support from management, and staff may experience a struggle of fitting use of a registry into a busy and complex everyday practice.

**Conclusion:**

The study highlights factors that may be important to consider when planning and implementing a new clinical quality registry within the field of cardiac rehabilitation, and is possibly transferrable to other fields. The results may thus be useful for policy makers, administrators and managers within the field and beyond. Targeting barriers and utilizing knowledge of facilitating factors is vital in order to improve the process of registry implementation, hence helping to achieve the intended improvement of care processes and outcomes.

**Electronic supplementary material:**

The online version of this article (10.1186/s12913-019-3940-5) contains supplementary material, which is available to authorized users.

## Background

The use of clinical quality registries (CQRs) is a common strategy to monitor and improve quality of services and care. A CQR, i.e. a structured collection of data on individual patient level within a specific area of health care, is aimed at monitoring and supporting health care in delivering high-quality services for the benefit of all eligible patients [1, 2]. A registry is intended to affect local practice by providing information about processes and clinical outcomes of care, indicating which aspects that need to be improved, and the feedback is supposed to facilitate quality improvement in the provider organisations [3]. In a national perspective, a CQR enables providers and stakeholders to evaluate performance and improvement against national level quality data [4].

While promising in theory, studies cast doubt on the potential of CQRs as tools in the improvement of care, pointing to several challenges. These include low perceived relevance of data, issues regarding how and when feedback is given, lack of know-how and resources for improvement activities, and poor collaboration between stakeholders [1, 4–8]. Furthermore, low data quality has been pointed out as a major barrier for use of data [1, 2, 9], and delays in data entry [10] and suboptimal coverage have been reported even in relatively mature registries [11].

Although there are multiple possible explanations for these challenges, they indicate problems with the implementation, i.e. the process of putting a CQR into practical use, from the initial startup to the continuous use of data for local and national quality improvement. Poor implementation has been identified as a common problem [12], resulting in suboptimal effects of new practices [13].

For CQRs too, proper implementation is crucial if they are to reach their potential as tools for quality improvement. To date however, implementation of CQRs has received scant attention in the literature. Within the field of implementation science, it has been emphasized that knowledge about context-specific determinants (i.e. barriers and facilitators) is important when planning initiatives to support implementation [14, 15]. While determinants for *use of data* has received some attention in CQR studies, there has been no detailed investigation of possible barriers and facilitators for *data collection and entry*, which constitute the fundamental first phase of CQR implementation. Although it has been highlighted [10] that participating healthcare providers are challenged by additional costs and workloads, and that delays in data entry are common, there is still limited understanding of what may actually help and hinder the process. In order to illuminate this, the purpose of this study was to explore how staff, entering data into CQRs, perceive the implementation process related to the registries.

### Setting

We studied the implementation of CQRs within the field of cardiac rehabilitation (CR), which is a structured set of post-treatment services aimed at improving health and quality of life for patient with heart disease [16]. CR has documented beneficial effects and is an important part of treatment in cardiovascular diseases [17–19]. Despite this, studies have documented a gap between the use of evidence-based recommendations for CR services and clinical practice [17, 18, 20–22]. As a strategy to overcome this gap, a number of CQRs for CR have been developed across the western world [10, 23] and further development of registries and data-driven improvement of CR has been called for [2, 22–25].

## Methods

### Two case registries

For the purpose of this study, the national cardiac rehabilitation CQRs in the UK and Denmark were used as cases. By choosing these registries, we were able to study implementation of a mature (the British) and a relatively new (the Danish) registry in two different countries and with different incentives for registry participation (voluntary and mandatory, respectively) [26, 27]. Funding and administration also differ. Similarities include scope and design of the registries, with variables being partly based on common European guidelines on CR, as well as largely similar data collection and data entry processes (Table 1).

**Table 1:** Overview over the two cases: national cardiac rehabilitation registries in the UK and Denmark

	The National Audit for Cardiac Rehabilitation (NACR)	The Danish Cardiac Rehabilitation Database (DHRD)
Country	The United Kingdom	Denmark
No. of inhabitants	65.6 million	5.7 million
Patient groups	Cardiovascular Disease	Coronary Heart Disease
Registry coverage	National (England, Wales, Northern Ireland)	National
Overall aim	Monitor and improve quality of outpatient* CR in the UK in order to improve the outcome for patients recovering from cardiac events	Monitor and improve quality of outpatient* CR in Denmark in order to improve the outcome for patients recovering from cardiac events
First launched	2005	2013 (fully operating 2015)
First annual report	2007	2016
Participation	Voluntary	Mandated by Danish law
No. of participating sites	224, hospitals and community	35 hospitals
No. of patient-level entries (annually)	Approx. 101,000	Approx. 6000
Governed by	Steering committee	Steering committee
Daily management	Administrative unit at the University of York. Team equivalent to 3,5 full time employees consists of a project lead, manager, training officer, data analyst and a secretary	The Danish Clinical Registries (http://www.rkkp.dk) The team consists of a manager, quality manager, epidemiologist, and a data manager, all of them with responsibility for DHRD as well as a number of other CQRs
Technical management	In cooperation with NHS Digital	In cooperation with external provider
Financing (except data collection)	The British Heart Foundation	Government (the Danish regions)
Financing of data collection and entry	Financed locally by each participating trust	Financed locally by each participating department
Data collection method	Electronic, web based Patient questionnaires are paper-based	Electronic, web based Patient questionnaires are paper-based
Data collected and entered by	Clinicians (mainly) or dedicated data administrators	Clinicians (mainly) or secretaries
User support opportunities	Training sessions, telephone, e-mail, written users manual	Telephone, e-mail, written users manual
Data linkage	No	Yes (The Danish Civil Registration System; the Danish National Patient Register; the Danish National Database on Reimbursed Prescriptions)
Patient consent	Opt out model	Not needed according to Danish law
Programme level data	Collected partly via database, partly via separate questionnaire (annually)	Collected via separate questionnaire (every third year)
Patient level data	Initiating event, treatment type, lifestyle, medication, demographics, pre-CR clinical outcomes and post-CR clinical outcomes, patient-reported measures	Initiating event, risk factor control, lifestyle, medication, demographics, pre-CR clinical outcomes and post-CR clinical outcomes, patient-reported measures
Feedback	Annual report; participating sites can get their own data via the NACR/NHS Digital database link (with login); programme level data available on general NACR webpage; specific requests on demand	Annual report; participating sites can get their own data (monthly updated) through regional clinical management systems (with login); specific requests on demand
More information available	www.cardiacrehabilitation.org.uk/nacr/ [27]	Zwisler et al. Clin Epid 2016:8;451–456 [26]

*Outpatient CR = In Denmark Phase II, in the UK core/Phase III: the initial 8–12 weeks of outpatient CR performed at hospitals and community level

Design and participants

The study was qualitative, based on semi-structured interviews aimed at gathering meaningful data about perceived barriers and facilitators to implementation and registry use among staff involved in collecting and/or entering data from sites using the two case CQRs [26, 27]].

An apparently similar intervention may be implemented and accepted in different ways in different settings [28]. Accordingly, several sites were included in this implementation study to capture diversity, which may lead to a broader understanding [29]. We sampled our informants with the aim of maximal variety, based on professional background, years of experience with CR, years of experience working with the CQR, type of hospital (university/non-university), geography (suburban, urban, capital) and organization of data entry (clinical staff and/or admin staff). In the UK, we chose to focus on England, as the countries in the UK are organized differently and England is the far largest country, also in terms of participating sites [30].

The informants were identified by contacting the coordinating nurse at the chosen sites by e-mail, explaining the purpose and format of the interview. They were asked to participate themselves and to invite a colleague with a different background and/or experience with the registry. All approached by an enquiry to participate agreed, except for one of the Danish (who had no time) and two of the English (who felt too unexperienced using the registry). Other clinicians with a similar background were then approached, and agreed to participate.

### Interview guide

The interview guide was based on theoretical and empirical knowledge about factors associated with successful implementation, including the Consolidated Framework for Implementation Research (CFIR) [31]. Inspired by previous knowledge, we strived to keep the interviews open to let the informants tell us as freely as possible about important aspects of implementing the registry seen from their point of view. Our definition of implementation as “the planned and systematic introduction of the database, with the aim to integrate the use of it in daily practice” was explained to all informants in the introduction. Following this, the opening question was “Tell us about your department’s implementation of [the registry’s name]”. If not mentioned, we probed for perceptions of the process which could illuminate hindering and helping factors. The subsequent questions were theory based and more specific.

The interview guide was pilot-tested, and a few questions were modified after four interviews, as the interviewers’ knowledge about the studied area evolved. There were Danish and English country-specific versions of the interview guide, as a few questions needed to be modified to suit the specific context (English version provided in Additional file 1). Supplementary field notes were written after each interview.

### Data collection

We conducted the interviews at the informants’ workplaces for their convenience, except for one interview, where the informant had to stand in for a sick colleague at the day for the interview and later chose to answer the questions in writing.

The interviews were conducted by the first and the second author, with one being the interviewer, introducing the interviewers and the study aim; the other observing, taking notes and making sure the questions in the interview guide were covered. Roles shifted between interviews. The first author has a theoretical /administrative background, with practical experience conducting interview-based research and working as an administrator for a CQR in another clinical field. The second author is a nurse with expertise in CR, working with the registry in practice, and a member of the steering committee for the Danish Cardiac Rehabilitation Database. Due to her clinical role, she knew some of the Danish informants beforehand, and in order to avoid bias, acted as the observer during these interviews. The combination promoted a good relation to the informants, as they had the clinical expertise and registry experience in common with one interviewer, counterbalanced through the naïve perspective on CR and registry use in practice by the other interviewer.

### Ethics

The study was approved by The Danish Data Protection Agency, REG-149-2015. No ethical approval was necessary according to laws, since it is not a biomedical study with inclusion of human material (Denmark), and did not include patients (the UK). All informants gave oral and written informed consent prior to onset of the interviews, including permission to audio record the interview. Data were treated confidentially.

### Data analysis

All interviews were transcribed verbatim and analysed using content analysis, inspired by the methodology presented by Graneheim & Lundman [29]. Content analysis has been described as a method for making replicable and valid inferences from data with the purpose of providing knowledge, new insights and practical guide to action [32]. In order to let the analyses reflect the informants´ perceptions as truly as possible we chose an inductive analysis approach, that is, with codes derived from the interview transcripts [33]. Three of the authors (first, second and last author) separately coded the interviews, and later discussed the codes, which had only few discrepancies, until reaching consensus for all codes. The codes were sorted and combined into subcategories and categories, constituting the manifest content (examples are presented in Additional file 2). The process of combining codes into categories was performed by the first and the last author, continuously reflecting on and discussing choices. Finally, a theme was derived, capturing the latent content of the interviews. Altogether, the categories and theme provide an understanding of staffs’ perceptions of the implementation process and illuminate possible barriers and facilitators for data collection and entry.

The Consolidated Criteria for Reporting Qualitative Research (COREQ) guidelines were used to guide writing of the manuscript [34].

## Results

### Informant characteristics

We interviewed 12 Danish and 12 English professionals, reflecting the multidisciplinary composition of the CR teams. They were either nurses, physiotherapists, dietitian or administrative staff, although the majority were nurses, as this is the main professional group collecting and entering data. Half of the nurses had a responsibility for coordinating the CR teams, and the other half were frontline staff members. No physicians were interviewed, as they rarely enter data. All but one of the informants were women. Informants’ experience with CR and working with the registry varied greatly (Table 2). The interviews were conducted in Denmark and England during the period September 20016-April 2017 and lasted between 15 and 47 min.

**Table 2:** English and Danish informants´ experience of working with cardiac rehabilitation and with the NACR and DHRD registries, respectively

	English informants	Danish informants
Experience with cardiac rehabilitation	< 1 to 23 years (median 15 years)	2–30 years (median 10 years)
Experience working with the registry (NACR in England; DHRD in Denmark)	2 months – 10 years (median 8 years)*	6 months - 3 years (median 1 year)**

* = Maximum possible time for NACR is 10 years ** = Maxium possible for DHRD is 3 years

### Struggling with practices

**Fig. 1:**
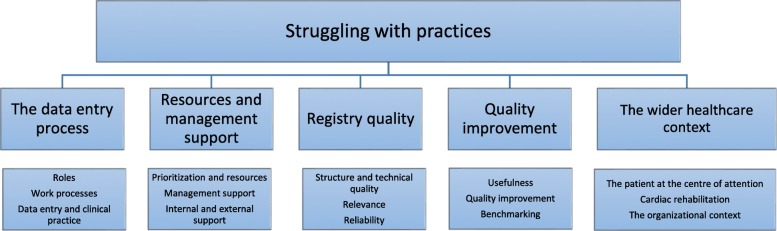
Theme, categories and subcategories in the study

One theme and five categories, each covering three subcategories, emerged from the analysis (Fig. 1). Representing the latent interview content [29], the theme ‘Struggling with practices’ concerns the multi-facetted challenges that may be part of adopting the CQR. It suggests that implementation of a CR registry is not a simple task of merely entering data into a reporting system, but rather a complex process that requires changes in practices and mindsets, as well as a sustained dedicated effort. This may be challenging in an everyday practice already faced with high workloads and competing changes to be made. Furthermore, the theme represents a more subtle struggle of getting acknowledgement for CR as an important part of cardiovascular treatment.

The categories underlying this theme reflect factors that the informants experience influencing the implementation and use of the CQR.

### The data entry process

This category covered the informants´ perceptions of organization of data entry processes and fitting it into everyday practice.

The implementation of the British registry (NACR) and the Danish registry (DHRD) had not received much attention, and some described using the registry as a “small thing”. Implementation efforts were found to be locally organized and clearly focused on getting access to the web-based system, data collection and data entry. Roles and responsibilities were allocated naturally, in many cases without formal appointment by management. Either the most interested staff members took on a leading or coordinating role themselves, or taking the lead was part of the expectations of being the local CR coordinator. Some Danish informants found that lack of management interference and lack of coordination within the team made implementation an individual responsibility. Most teams had found it “natural” that the clinician seeing the patient – thus collecting the data – also was to enter data. Some perceived it important to have clinical expertise to manage the task properly. However, at a few sites, both in England and in Denmark, the task of entering data was passed on to administrative data entry staff, or to a few of the clinicians instead of all team members. The aim was to save precious clinician time, and to specialize and divide work tasks (administrative versus clinical).

In both countries, collecting and entering data was an extra workload that was to be fitted into everyday practice. The nurses, who collect and enter the majority of the data, found this more or less time-consuming and some perceived it as a cumbersome task. The physiotherapists and dietitian on the other hand, who have less extensive data forms to fill out, perceived data entry as rather quick and straightforward. Regardless of professional role, most found it necessary to register data onto paper-based records first as focusing on the computer screen while the patient is present would disturb patient contact. Only at one English site, direct online entry without intermediate paper records was reported, but it still took place after the patient visit. Furthermore, locally or individually invented notes/lists were used to keep track of patient follow-ups at almost all sites. The informants found this necessary because the registries were not designed to flag patients due to specific follow-ups, although such data may be required by the registries.

The informants found it – often an experience gained along the way – as a clear facilitating aspect to make data entry part of everyday workflow and enter the data immediately after the patient visit, or at least the same day. By doing this, data are fresh in memory, and the task seems more relevant.

What I think has worked well is that data entry has been tied to existing routines. Because it… makes you remember it much easier. And I also believe that’s why we get so many patients entered, as we do. It’s tied up to existing routines. (DK8)

Some sites reported struggling with getting data entered. Here, the data collection and/or online data entry was not an integrated part of daily work processes, but rather a duty performed when time permitted or when extra resources were allocated, for instance before the annual reporting deadline to the registry. This was described as a very time consuming and negatively associated task.

The task of collecting data may require redesign of practice in order to be able to fill out the registry’s minimum requirements, for instance introduction of new routines such as weighing the patients or screening for depression using a recommended screening instrument. Furthermore, collecting patient-reported data by questionnaires and keeping track of follow-ups require attention and new routines. Both data collection and -entry necessitate collaboration and division of tasks within the multidisciplinary team. Some informants found that data collection structured the conversation with the patient, whereas others did not find any positive influence on daily routines.

### Resources and management support

This category included issues related to resources and prioritization, support from management, and support within and external of the CR team.

Although working with the registries was described as more or less time-consuming, only few English and Danish sites had received extra resources for the task. Time must thus be found elsewhere, mostly reported taken from the dedicated patient time. Another solution was to register only the minimum required variables, although some found this unsatisfactory, as they believed output data would be more interesting if most/all fields were filled out. Nonetheless, most sites in both countries prioritized the task of collecting and entering data highly, either because they supported the idea of a registry and wished to contribute, or because reporting was mandatory (Denmark). A few informants did report low priority of the task, even in Denmark despite the fact that reporting is mandatory. This was mainly because of low staffing or because the registry got a back-seat to other high priority activities. Some of the informants felt bad about this as they knew it was a “must-do task” which they dutifully wished to fulfil.

Nearly all informants reported low levels of knowledge, interest and support from management in the initial phases of registry implementation, where data was collected and entered. A “silent accept” was experienced in several sites in England where the uptake of the registry was bottom-up driven by engaged clinicians, and management for instance allowed staff to attend training. While some reported that this lack of interest remained even when feedback data started coming and results were getting published, others experienced that the management were very interested in data and results.

I met a lot of resistance from my manager who said we are spending clinical time inputting and gathering data but we’re getting no feedback. […] And now that manager has changed her mind about the value of NACR and thinks that the information is brilliant, because now the commissioners want to use it as their reporting tool. (UK7)

In England, most of the staff involved in the registry in its early years had received formal training under the auspices of the registry administration. The new users had on the other hand not had training, and relied on written guidance, or if applicable, colleagues. In Denmark, in contrast, no formal training had been offered at any time, although some of the coordinators had participated in start-up meetings. As the DHRD was relatively new, most of the informants also had had no colleagues to teach them about the system, which meant that they had to learn the system by themselves as they went.

And it was learning by doing, and that’s the way it was. […] I have not been introduced to anything what so ever, so it’s jumping right into it, and find out what we are supposed to inform about, and what we are not to inform about, and what we are supposed to write, what we are not supposed to do, and… Well. (DK9).

In England, the users experienced very good help from the national administration office, although some of the most recent new users did not know of the support opportunities. This lack of awareness was also seen among some of the Danish informants, who did not know of any external support opportunities, and therefore relied on colleagues or merely resigned receiving help. Of those who did know whom to contact for help, experiences were mixed, and in particular, a lack of action on functional problems in the registry was reported. Among the very few who had insight into the registry organization system, this was explained as inertia within the system. The lack of action was discouraging.

Use of formal and/or informal networks was common among the more experienced staff, both for asking questions and for discussions. The more inexperienced staff did not have this opportunity, however, as formal networking opportunities were rarely offered to them, and as new in the field they had no informal networks in the CR community.

Communication from national administration offices to users about the registries was perceived a problem both Denmark and England, however rarely in the latter. This meant that important information may not reach the relevant users; for instance, the physiotherapists at one Danish site had not received information about re-launch of the registry and thus had not entered any data even after one year, and annual reports did not reach the clinical staff.

### Registry quality

This category covers structure and technical quality of the registries, and the relevance and reliability of data.

The structure and technical quality of the registries was important for their usability. Most found it easy to enter and navigate both the NACR and the DHRD, and the English informants described that the user-friendliness of the NACR had improved a lot over the years. However, meanings were divided both within and across countries concerning the registry structures, where some perceived it fairly adapted to the patient pathway, while others found it challenging to enter the relevant data due to the perceived mismatch. In DHRD, data linkage to external registries had been established to save time in data collection and entry. However, due to delays in the external registries and technical problems, the users experienced missing data and problems with the quality of data pulled into the DHRD, which was a source of remarkable frustration.

*What I think more about is that is it poor data catchment. Really poor. There are many things it doesn’t capture; medicine, diagnoses… So there are things it catches where you go ´What? That’s not true´. Everyone actually thinks it’s a little annoying to look at something which isn’t correct* [but we have been told by management not to correct this, as it is not marked as mandatory variables]*. And you’d think, what can they use this for? If data are not correct or even missing? And I think we use a great deal of energy on speculating about… is it wasted resources, this, or what is it supposed to be used for? I think this is most frustrating. Yes, it is… (DK9)*

Timesaving functions in the registries, e.g. body mass index calculators or the possibility to copy a summary of data into the electronic health record, were on the other hand highly appreciated and encouraged use of the registry.

The perceived relevance and reliability of data were reported important for the motivation to use the registries. The informants found the chosen variables relevant. However, they did not cover *all* the important aspects of CR, and most would like the variables (which are process and clinical outcome measures) to be supplemented by variables that capture psychosocial values, as this was expressed as important outcomes when working with CR. The English informants appreciated the possibility to adapt the choice of variables to match local practice, as only few variables were mandatory. However, some found it necessary to supplement the NACR with local databases, as those were easier to fit with local demands for data.

In both countries, but particularly in Denmark, users experienced ambiguity in the variables. This caused frustration in the data entry phase, and in addition, a pronounced distrust in data especially among the Danish informants.

Some of it is open to interpretation and sometimes I have scratched my head and ‘does it mean this or does it mean that’ and I’ve input it one way and colleagues may have put it differently (UK5).

… the data that are being entered, you can write anything. And it is totally dependent on how you… view it yourself. So I don’t think it is […] valid. […] You can’t use it for anything at all. So I actually think it is […] a little demotivating. (DK12).

### Quality improvement

In this category, we included both beliefs and actual experiences of the usefulness of registries for quality improvement.

Insight in feedback data was found to vary greatly, both within an in between the two studied registries. In England, where feedback data had been published for years, most informants had at least had a glance at feedback data, and some knew data well. In contrast, most Danish informants had neither received nor sought feedback data from the relatively new DHRD. Some had studied data, although it was found to be partly difficult to understand.

The actual *use* of data varied. English coordinators used data to provide productivity data to local commissioners, and a few (primarily English) had used the data to put pressure on their management to invest more in CR and found this very useful. In general, there was limited awareness of the fact that data were gathered to aid local quality improvement. Rather, it was believed to be used for research. Some knew data were supposed to be used for local quality improvement but realized that this requires time and competences and that neither are present in most CR departments.

If data is to be useful, it needs to be reviewed, discussed, and outcomes need to be considered in relation to own practice. When short staffed, this type of work does not get done. Our Heart Failure colleagues have used our data to present the numbers of heart failure patients being offered Cardiac Rehabilitation. But from the management of our service, we have not yet really used NACR to change practice. (UK11).

Some stated that quality improvement takes place anyway, but not based on registry data. Among English informants, some described to be motivated to use NACR by seeing improvements in the registry data. There were however staff in both countries who did not find the database useful at all. In particular, some of the Danish informants were highly sceptical of using data, as they had a great distrust in its validity. Following this, they regarded the resources spent on data collection and entry as a waste of time.

Informants in both countries supported the *idea* of a registry as this meant a possibility to improve quality of CR for the benefit of the patients. It was also believed to be an opportunity for acknowledgement of CR in a wider sense, and to highlight the extent and importance of the work that staff put into daily practice.

…everyone needs an audit wherever you are, there has to be something to acknowledge how many patients coming in, why and how it’s working, so we knew there had to be audit. (UK12)

Some informants, both in England and in Denmark, valued the possibility to compare results of their own department to others, and stated that this could potentially provide learning opportunities. Others did not appreciate the benchmarking, as it added a competitive element.

### The wider health care context

This category covers issues of the context, meaning the organizational and wider environmental factors that may affect implementation. It includes the patient, CR as a clinical field, and the wider healthcare context.

The patient was clearly at the centre of attention among the interviewed clinicians. The use of a registry sometimes supports this focus, for example the abovementioned structuring of the conversation with the patient and the prospect of receiving acknowledgement for CR. Others described the registry as a disturbing element, forcing them to use precious clinical time on data entry instead of on the patient. As patients are individuals, their pathways sometimes diverge from the norm and were thus difficult to fit into the registry, and patients may not wish to respond to questionnaires required to fill out the registry. As a clinician, one may have to choose between spending time on issues that are relevant to the individual patient versus working through all variables necessary to fill out the registry.

Both the English and the Danish informants found themselves faced by growing administrative workloads in general, making it even more difficult to find time for the registries. A few of both the English and Danish clinicians expressed healthcare as increasingly being a business driven model, where the registries and the focus on documentation and reporting was an integrated part. For some this was already the new reality, others realized that they would have to adapt.

You just take it as part of the workload, it’s what you do. Audit and information gathering now is routine in health care and it’s right. (UK8)

In the heart failure clinic, registering data has been part of the job for years. But it isn’t for cardiac rehab nurses. Therefore, it’s another culture, that one is… that it is part of the job to enter data into a registry. (DK6).

Yet others did not express awareness of culture issues and were in general opposed to the increased documentation.

Among the Danish nurses, some expressed fear of their professionalism being set aside, as they believed management focused too heavily on following registry requirements instead of clinical experience.

Some of the English nurses compared the NACR to other cardiac CQRs with economic incentives for participating, noting that this seemed to make a difference for prioritization at management level. The fact that participating in NACR recently had become part of a certification programme for CR had gained interest among some commissioners. In Denmark, the informants were generally unaware of laws or national guidance that mandated or recommended data reporting, but did know that data reporting was non-optional.

## Discussion

This study of real-life implementation experiences among professionals taking active part in registry usage documented a range of experiences and beliefs. Many were found to be similar across England and Denmark, but there were also a number of differences both within and between countries. Although these experiences and perceptions were not always explicitly expressed as barriers and facilitators for implementation, they may to some degree of certainty be interpreted as such. In the following, we thus highlight and discuss some of the key findings while assessing them as barriers and facilitators for implementation (for an overview, see Table 3). Since many of our findings can be related to the Consolidated Framework for Implementation Research (CFIR) [31], which identifies a number of determinants of implementation divided into five domains, we let the CFIR domains provide a structure for the discussion.

**Table 3:** Selected key findings assessed as barriers and facilitators for clinical quality registry implementation, organized by domains in the Consolidated Framework for Implementation Research (CFIR)

CFIR domain	Barriers	Facilitators
Intervention characteristics	Practice changes often required but not foreseen. Ambiguity of registry variables. Poor registry design/functioning with regards to e.g. patient follow-ups. Poorly functioning data linkage. Typing on computer screen diverts attention from patient.	Continuous development and adjustment of registry function and content, as needed. User-friendly layout and design.
Inner setting & Outer setting	Lack of management support in data collection and entry phase. Lack of incentives.	Management interest in output data (results). Feedback data regarding local use of resources and local quality. Use of registry included in cardiac rehabilitation certification programme. Mandated participation in registry. Results part of national quality indicators. The prospect of improving patient care and raising acknowledgement for cardiac rehabilitation. A culture of data reporting.
Process	Lack of formal planning of implementation process. Implementation a responsibility of the individual clinician (or few clinicians). Lack of support and clarification.	Training and support of users.
Characteristics of individuals	Lack of knowledge about purpose of the registry. Lack of know-how and resources to use data for local quality improvement.	Local registry advocates/ champions.

The CFIR domain *Intervention characteristics* emphasizes the necessity of adapting a new intervention to the setting, except for its core components, which are essential and indispensable elements of the intervention [31]. Our finding that data *collection* require redesign of practice at some sites, primarily Danish sites because of the larger number of mandatory fields, is therefore interesting because it indicates that not only is use of the registry to be fitted into practice, practice processes are also influenced by the registry. This may be a positive effect if it contributes to improving quality or limiting unwanted variations in the provision of care, but seen from an implementation perspective, it adds to the complexity. Most informants did not seem to be aware of the necessity of these practice changes until being in the process of implementation. These aspects highlight that registry implementation is more than merely registering data into a database and hence, a more complex task than apparently first expected. To our knowledge, this aspect has not previously been described in CQR implementation. Previous research underscores that foreseeing necessary practice changes and including them into an implementation plan contribute to successful implementation [35].

Another aspect of the intervention characteristics domain in CFIR is the ‘design and quality’ of the registries, and in our study, three main issues emerged. Firstly, the ambiguity of variables was a source of frustration, and both real and perceived effects on data quality is to be taken seriously, as it affects users´ motivation to enter data, and because high data quality is fundamental for the use of data for quality improvement and research. Secondly, the fact that all informants but two reported using locally invented registration forms/lists to keep track of data and patients and to retain focus during the patient encounter indicate that there is room for improvement of the registries’ user-friendliness to better fit multiple different practice processes, and thus facilitate registry use [1, 4]. This need is underscored by the finding that use of paper-based data collection may introduce opportunity for data error in the transfer to the web-based platforms [36]. The third aspect of design and quality is data linkage, which has often been emphasized as a great advantage of CQRs, saving precious clinical time by avoiding double entry and improving data quality [4]. Although data linkage was supposed to be a facilitator for registry use in the Danish registry, the poor execution seem to have had the opposite effect; to a high degree creating a barrier because of the frustrations and demotivation it caused. This emphasizes the importance of assessing the quality of the source registry and thorough testing before data linkage is implemented [4]. Altogether, the issues related to ‘design and quality’ stresses the need for registry organizations to secure sufficient resources to continuously react on and remedy flaws, since such agility appears to facilitate continuous support of a registry.

The next two CFIR domains are *inner and outer setting* [31], which deal with structural, economic, political and cultural contexts in which the implementation takes place. In line with CFIR suggestions, we regard the lack of management support in the data collection and entry phase as a major barrier for implementation. In addition to the immediate challenge of not prioritizing and allocating necessary resources, it may also indirectly affect the implementation climate because of the lack of active interest [31]. In contrast to the lack of interest and support in the data entry phase, the managerial interest in output data spurred data entry, which mirrors previous Swedish findings [9]. It was beyond the scope of this study to examine managers’ perceptions of CQR implementation, but our findings point to that this may be an important focus for further study.

‘Incentives’ are another part of the settings domains in CFIR, which seemed to play an important facilitating role in our study. In England, receiving feedback reflecting local quality of care and use of resources emerged as an incentive to voluntary join NACR in its first years, and although still important, now seem to be co-working with another incentive: certification, to encourage participation in the registry. In Denmark, the external policy incentive of mandatory participation did not guarantee full data entry, as there were reports of differences in local prioritization, reflected by coverage data in the DHRD annual report [37] and also mirrored in Swedish findings [11]. Although our study may provide some explanations, not least the overall limited focus on securing implementation, it could be a combination with a lack of penalties/incentives on a national level. Notably, a new external incentive was introduced in 2016 as results from CQRs were included as a major national and local healthcare quality indicator [38], and this is likely a reason for the Danish informants´ reports of managements´ interest in performance data. However, based on our data, it seems that there is an imbalance between the strong focus on output data and the relatively little focus on the processes of collecting and entering data and using it for local quality improvement. Moreover, although incentives related to audit and feedback, national legislation, and programme certification or other reimbursements have been suggested to be more effective than voluntary participation [10, 39], improving patient care and raising acknowledgement for CR emerged a less tangible but strong incentive. This drive could explain some of the within country differences in participation, and could possibly be activated more explicitly as a strategy to improve participation.

The informants´ expectations that documentation per se will lead to acknowledgement of CR is mirrored in a recent report by the World Health Organization, where use of national audits to document provision, quality and outcome of rehabilitation services is suggested to raise awareness among for instance policy makers [40]. In a wider perspective, the motivation to document data in a registry reported by our informants seem to be reflecting an institutionalization of CQRs [41], as part of the quality measurement enterprise permeating healthcare [42]. These expressions about a culture of data reporting may be important in an CQR implementation perspective, as it – as suggested by e.g. CFIR – can explain why efforts that are targeted at more tangible aspects fail to work, and in the cases of the present study can provide an additional explanation to within-country differences in implementation experiences.

The last two CFIR domains are *individuals* and the *implementation process*. Individuals are those who are involved in the intervention and/or the implementation process, which in turn is the active change processes aimed to achieve use of the intervention [31]. In our study, these two domains were closely related. Very little formal planning of the implementation was reported in either of the studied countries, which, combined with the lack of management involvement, made implementation a responsibility of the team or even individual staff members. In this situation, the capacity of highly engaged teams or individuals played a vital role in facilitating the implementation. The important role of such champions has been emphasized in numerous implementation frameworks, including CFIR.

Besides engaged individuals and teams, the training and support by the NACR registry administration clearly facilitated data entry, whereas the lack of training and lower level of support experienced among DHRD users in Denmark interestingly did not seem as a distinct barrier for getting data entered. This points back to context, as it is likely to be an effect of the mandatory participation. In addition, it could be indicating that the computer literacy in general is high and that the system has a user-friendly design, which has previously been indicated as facilitating implementation [1]. While some may argue that this suggests that training and support is not necessary as part of CQR implementation, the findings must be seen in perspective of the issues with data quality that became evident in later stages of registry use, when the users – along the way – found out that there is ambiguity in some registry variables and that they may be filling things out incorrectly. Here, lack of support and clarification was a barrier, annoying users. This, in turn, affected the perceived trustworthiness of the registries and demotivated the users. Although a few NACR users mentioned issues with data, this problem was not prominent in England, suggesting that the decade long continuous development of the registry and high support level is making a difference. Some of the differences we found between NACR and DHRD are thus likely to be due to registry maturity and administrative resources.

Besides data entry issues, not all informants were aware of the purpose of the registries, and/or were lacking resources and know-how to use data, and overall, very few of our informants reported examples of actual use of data to improve care. Ensuring adequate resources and competencies of the staff has been emphasized both to ensure high-quality registry data [1, 36] and use of data for quality improvement [9], and this focus should be continuous to take into account e.g. well-trained staff that leave and new staff that should be trained [12, 36]. However, it is evident that front-line staff and managers cannot stand alone; all stakeholders have important roles to play in order to secure successful use of the registries [31, 43].

Overall, the many similar experiences among users of the two CQRs suggest that there are some common barriers and facilitators of using a CQR for CR. They may be common for two reasons: firstly, because they may be generic to implementation [44], as indicated by their presence in compilations of previous implementation studies such as the CFIR. Secondly, it indicates that there may be aspects of using CR CQRs that are specifically tied to this quality improvement tool per se [4, 44], and therefore present across settings. The dissimilarities on the other hand seem to be explained in part by differences in registry administration, design, and incentives. The relative maturity of NACR compared to DHRD creates different challenges and opportunities for users and administrators, as different implementation phases require different considerations [12]. The dissimilarities were furthermore interpreted as reflecting differences in local and nationwide healthcare organizations and culture, and individual characteristics of informants.

### Strengths and limitations

We consider the design with two international cases a real strength, adding valuable insights beyond the single registry and widening our understanding of potentially important factors to consider in similar implementation situations [45]. To further enhance trustworthiness, we strived to include informants with different roles and experiences to give a broad perspective on possible barriers and facilitators for implementation [46], and kept on until we got no new information from the interviews [47]. In spite of our efforts, there may be experiences that were not covered, and the questions may have focused on certain aspects while leaving out other possibly important aspects. Use of broad and open ended questions were intended to minimize this restraint on subjects [48]. Nevertheless, qualitative findings are by their nature context and case dependent [49], and transferability to other settings should be judged by the reader [29].

Researcher preconceptions may influence both the data collection and analysis, and is therefore important to describe. The primary investigator had an a priori expectation that implementation of the registries often would not receive much focused attention and that it would be challenging for staff to manage in a busy everyday practice, resulting in poorly implemented registries. To limit influence of such preconceptions, we used researcher triangulation [49], where the two co-analysts had other backgrounds and thus analysed data from different perspectives. This promoted valuable discussions between the co-investigators that we believe strengthened our insight and thus our categorization of data, hence enhancing the quality of the analysis [29, 49].

Because we included two countries in this study, interviews were carried out in two languages, where English is second language for both interviewers. Despite a good knowledge of English, there may be things that we did not understand as subtle as we did with the Danish interviews, limiting e.g. the flexibility to follow up on unexpected information during the interviews. To remedy possible limitations in our understanding of the oral language, transcriptions were carried out by experienced native English transcribers with a good knowledge of the English healthcare system and clinical registries, and they were also asked to clarify the meaning of a few idiomatic expressions [50].

## Conclusion

This two-country, real-life study points to a range of factors that may support or hinder the implementation of a CQR for CR according to the healthcare professionals´ perspectives. Implementation can be a more complex process than first expected and staff may experience a struggle of fitting use of the registry into a busy and complex everyday practice, often with little support from management. The findings are relevant, because they emphasize that a registry is not implemented by merely launching it, and that getting high-quality data into a registry requires a dedicated, sustained effort that involves not only staff but all stakeholders. The study thus highlights the importance of acknowledging the challenges of CQR implementation and of supporting it by applying appropriate, if necessary multi-facetted, strategies at multiple levels. Results may be important to consider for all stakeholders involved in planning, launching or implementing a new CQR for CR or in related clinical fields, or for those involved in improving use of an existing registry.

## Additional files

Additional file 1: Interview guide, English version. Table showing the topics and questions of the interview guide. (DOCX 27 kb)

Additional file 2: Example illustrating the coding process of content analysis. Table with examples from three different English interviews. (DOCX 13 kb)

## Abbreviations

CFIR: the Consolidated Framework for Implementation Research
CQR: Clinical Quality Registry
CR: Cardiac Rehabilitation
DHRD: The Danish Cardiac Rehabilitation Database
NACR: The National Audit of Cardiac Rehabilitation (UK)
