# Supplementary material for: “Struggling with practices” – a qualitative study of factors influencing the implementation of clinical quality registries for cardiac rehabilitation in England and Denmark
Source: BMC Health Serv Res. 2019 Feb 6;19:102. doi: 10.1186/s12913-019-3940-5 (PMC6366013; doi:10.1186/s12913-019-3940-5)
Supplement: Supplementary file 1 — Interview guide, English version. Table showing the topics and questions of the interview guide. (DOCX 27 kb) [file 12913_2019_3940_MOESM1_ESM.docx]

# Additional file 1: Interview guide, English version

| **Briefing / introduction** | **Information (bullets)** |
| --- | --- |
| Presentation of interviewers and purpose of the interview  Frame of the interview  Informed consent  Presentation of informant:  Background  Role concerning NACR | Who are we  Purpose  Roles  Our focus is the implementation of DHRD/NACR. We define implementation as *the planned and systematic introduction of the database, with the aim to integrate the use of it in daily practice*  Timeframe  Recording  Confidentiality/anonymizing  Clarification of questions  What is your professional background and what is your role in relation to implementation of NACR?   - How did you get this role? |

| **Implementing the database in your department** | **Interview questions** |
| --- | --- |
| 1. Procedures / what happened | Tell us about your department´s implementation of NACR (*probes*: who, when, what did you and your colleagues do? Why?)   - Please describe what worked well –and why/what made it work well/ facilitated the process? - What worked less well? Why / what made it work less well /acted as a barrier in the process? |
|  | Could you briefly describe the model (process) you have chosen for entering data into the database?   - Why did you decide – or end up – doing it this way? - To what degree does use of the database give you an extra work-load?   Which factors influenced the decision to implement NACR in your department? |
| 1. Division of work and cooperation | Who was / is formally appointed with responsibility for implementing/sustain use of the NACR as coordinator, project manager, team leader, or other similar role?   - Has someone (or a team) outside your organization been helping you with implementing NACR?   To what extent do you network with other health professionals outside your setting regarding NACR?  Has anything been done to encourage individuals to commit to use the database?   - Has this been successful? |
| 1. Support and interest from management | What level of support have you experienced from leaders at your department/ hospital?  Have your department set goals related to the implementation of the database?   - Are these goals monitored for progress? |
| 1. Organizational activities / support | Now I would like you to think about the organization of the work using the NACR, for instance division of work, planning the data entry process, follow-up on results. What possibilities are there to get support to the process of using the database?  What kind of support is available to help you use the database? E.g. online resources, toolkit, “help-desk”/ administrations office, training/courses and the like   - Is it unambiguous what to register? |
| **Feedback from NACR** | |
| 1. Received feedback | Do you receive feedback reports about the implementation or the intervention itself?  Apart from the annual report, do you receive any other kind of feedback? Oral or written. (From network, external partners, leaders, patients, colleagues…) |
| 1. Use of feedback | How – or in what ways – do you / your department use the feedback you receive?   - Did your colleagues receive the same feedback / have they seen it? - *If they use feedback*: Who is working with the feedback? - *If they use feedback*: To what degree do you experience support from you colleagues in the improvement work?   Does the annual report or other feedback help you assess progress towards implementation or treatment targets?  What is your perception of the feedback you or your department get?   - Quality - Relevance - Wishes for future feedback   Does the database capture what you think is important in cardiac rehab? |
| 1. Feedback strategy | What do you think of data from your local department will be published openly? |
| **Importance of the database for daily clinical work** | |
| 1. Other projects and activities | To what extent might the implementation of the database take a backseat to other high-priority initiatives going on? |
| 1. Relevance for clinical practice | Which effect has NACR had on daily practice?  What is your opinion of the indicators that are chosen for NACR?  In what ways do you think the NACR will affect cardiac rehab in the future?  Overall – do you believe the database is optimizing cardiac rehab for the benefit of cardiac patients? |
| 1. Overall benefit | Overall, do you think the work with NACR is worth the effort? |
| **Debriefing / end of interview** |  |
| Wrapping up  What will happen now | We are about to be finished with the interview…  Is there anything else you would like to tell us / anything to add?  May we contact you again if we have any additional questions / details?  Information about writing of paper and publication |
