# Supplementary material for: “Struggling with practices” – a qualitative study of factors influencing the implementation of clinical quality registries for cardiac rehabilitation in England and Denmark
Source: BMC Health Serv Res. 2019 Feb 6;19:102. doi: 10.1186/s12913-019-3940-5 (PMC6366013; doi:10.1186/s12913-019-3940-5)
Supplement: Supplementary file 2 — Example illustrating the coding process of content analysis. Table with examples from three different English interviews. (DOCX 13 kb) [file 12913_2019_3940_MOESM2_ESM.docx]

# Additional file 2: Example illustrating the coding process of content analysis

Examples from three different English interviews.

| **Text unit from transcribed interview** | **Code** | **Subcategory** | **Category** |
| --- | --- | --- | --- |
| Interviewee: I [asked a colleague] the other day when I had to put in the initial contact data, I needed to refer to the administrator and a colleague of mine, the Band 7 nurse she was able to give me more information about what needed to be filled in because I didn’t know, like the GP address and whatever, the patient address and all that business. | Internal support | Internal and external support | Resources and management support |
| Interviewer: What kind of support is available to help you use the database?  Interviewee: Just speaking to colleagues really. I’m not aware of any other resources or anything like that. | No knowledge of external support | Internal and external support | Resources and management support |
| Interviewer: To what extent might the inputting of data into the database take a back seat to other high priority tasks?  Interviewee: It doesn’t. I see the patient, I enter the data and that’s it. It’s done straight away […] | Data entry part of routine | Work processes | The data entry process |
| […] over the last couple of weeks we’ve been a bit quieter than we normally would be, so I’ve had the time [to enter data]. | Enters data when time left | Work processes | The data entry process |
| Interviewer: Have you ever considered that the nurses would do the registering?  Interviewee: We just don’t have time. When we originally started we were doing more of it and we did consider should we be inputting the data […] it was basically a time thing because [our admin staff] does X hours a week with us and we don’t have to think about that and we can concentrate on what we do and all the patient stuff, erm, so yes it was originally considered and it was decided that it wasn’t appropriate for us to do other data inputting. It wasn’t a good use of our time and sort of expertise […] | Work division nurses vs. admin staff | Roles | The data entry process |
